# Patient perceived value of teleophthalmology in an urban, low income US population with diabetes

**DOI:** 10.1371/journal.pone.0225300

**Published:** 2020-01-09

**Authors:** Rajeev S. Ramchandran, Sule Yilmaz, Evelyn Greaux, Ann Dozier

**Affiliations:** 1 Department of Ophthalmology, Flaum Eye Institute, University of Rochester Medical Center, Rochester, NY, United States of America; 2 Department of Human Development and Counseling, Warner School of Education, University of Rochester, Rochester, NY, United States of America; 3 Department of Biology, College of Arts and Sciences, University of Rochester, Rochester, NY, United States of America; 4 Department of Public Health Sciences, University of Rochester School of Medicine and Dentistry, Rochester, NY, United States of America; Chinese Academy of Medical Sciences and Peking Union Medical College, CHINA

## Abstract

Dilated eye exams are the standard of care to detect advancing, vision threatening, but often asymptomatic retinopathy in a timely fashion, allowing for vision preserving treatments. Annual exam rates are suboptimal, especially in underserved populations. Although teleophthalmology programs tremendously improve annual exam rates in low income/under resourced settings, widespread adoption is limited. Using a mixed methods approach, three focus groups and individual interviews were conducted for patients with type 2 diabetes (N = 23) who had a teleophthalmology exam or a dilated eye exam. A survey and discussion assessed patients’ perspectives and value of teleophthalmology, including willingness to pay (WTP). Financial, transportation, and motivational barriers to obtaining an annual dilated eye exam were identified. Patients greatly valued having primary care (PC) based teleophthalmology for its convenience and ability to detect disease to allow for timely treatment and would recommend such a service. Although their WTP was at least the amount of their usual copay, cost was universally cited as a concern. Having a conveniently offered PC based teleophthalmology exam was valued. Educating patients on the value and costs of having such exams may be helpful to encourage informed discussions on eye care, especially in low income, underserved populations. Our study is among the few to provide insight on the value and perceptions of teleophthalmology in US low income, urban minority populations needed to help increase uptake of this innovation. Using surveys followed by facilitated discussion allowed for richer and more varied responses.

## Introduction

Affecting almost 10% of the US population, diabetes mellitus is a growing pandemic, with a third having diabetic retinopathy, the leading cause of blindness in the working age population [1]. Annual dilated eye exams are the standard of care to detect advancing, vision threatening, but often asymptomatic retinopathy, in a timely fashion [2], allowing for treatments that preserve and improve vision [3, 4]. Unfortunately, at best, only 60–70% of those with diabetes have an annual dilated eye exam. This percentage averages between 25–35% for low income populations in under resourced settings [5, 6].

Teleophthalmology is the innovative utilization of nonmydriatic fundus camera-based retinopathy examination in non-eye care settings, including primary care offices, using eye doctors to remotely grade images and recommend appropriate follow-up eye care. Although such programs have tremendously improved annual examination rates for retinopathy in low income and under resourced settings [7–9], widespread adoption of such examination technology and processes have yet to occur. Research on patients’ attitudes about teleophthalmology is limited. Understanding how patients perceive the value of using teleophthalmology programs to screen for retinopathy and assess vision in non-eye care settings is important for developing successful programs and increasing its adoption [10, 11]. Qualitative assessment of patient experiences with teleophthalmology through focus groups and interviews allows for improved design and implementation of such programs by understanding local consumer needs [12–14].

Conducting focus groups and qualitative analysis to elicit patient experiences and obtain candid perspectives of their health and health care yield richer insights into local community beliefs that influence adoption of health practices than quantitative questionnaires or surveys alone [12–20]. Cost and access have been identified as the two main barriers of obtaining dilated eye exams from focus groups assessing the knowledge, attitudes, and beliefs of patients with diabetes in urban and rural settings in the US [16, 19, 20]. None of these studies addressed the use of teleophthalmology to examine eyes for diabetic retinopathy.

The majority of work evaluating patient experiences with teleophthalmology has focused on international populations using quantitative surveys [21–27]. One qualitative study from the UK found that participants value teleophthalmology if they understand why it is being recommended and if it is convenient and accessible by safe transportation [28]. One of the few studies to assess teleophthalmology users in the US noted that patients may not understand the reason for these examinations [29, 30]. A survey of US Veterans with diabetes also found that convenience was a key factor in favoring teleophthalmology. However, this cohort had not actually experienced teleophthalmology [31]. A recently published study identified barriers and facilitators of teleophthalmology among rural, white Caucasian patients in Wisconsin who had experienced this type of examination found that the convenience of teleophthalmology was a key facilitator, whereas not knowing enough about teleophthalmology was a key barrier for having such an exam [30]. Our study investigates how patients value having a teleophthalmology examination offered in urban US primary care provider (PCP) practices serving low income, minority patients. We include the perspective of those who have and who have not undergone such a teleophthalmology exam using qualitative analysis.

## Methods

This study was approved by University of Rochester’s Research Subjects Review Board as an exempt study (RSRB00065090). The ethics committee approved the verbal consent procedure and did not require written consent due to the nature and the activities of the study. All participants provided informed verbal consent for their participation and for the audio-recordings during initial phone contact to schedule an interview or a focus group. Participants received $25 cash for their participation in the focus group or interview as well as bus tokens as needed for transportation.

### Setting

Two primary care settings serving low income, largely minority, inner city populations in Rochester, NY implemented teleophthalmology programs in conjunction with a local University-based ophthalmology department in 2013 and 2015, respectively. One clinic was in a health system outside the University system. This clinic was hospital based, with approximately 2100 patients with diabetes. The other clinic, owned by the University, was located in a neighborhood setting and had about 500 patients with diabetes. The teleophthalmology program used a Zeiss Visucam NM PRO (Carl Ziess Meditec, Dublin, CA, USA) nonmydriatic fundus camera in the hospital clinic and the Topcon NW400 (Topcon Medical Systems, Inc, Oakland, NJ, USA) nonmydriatic fundus camera in the neighborhood clinic to take three standard fields and one anterior segment photo of each eye. Both clinics used Snellen visual acuity charts to examine patients with diabetes for vision loss without dilating their eyes. Patients without a documented eye exam (per HEDIS criteria) were identified and slated for a teleophthalmology based exam either at their next PCP visit or were scheduled for a diabetic management nurse visit where they received the teleophthalmology exam.

A patient care technician or nurse obtained identifying information, assessed visual acuity, and took monoscopic digital photos of the eye. The latter were uploaded to a secure cloud-server. After the images were read by a single ophthalmologist (RSR) from the university eye institute within 1-day, electronic reports describing presence of any disease and visual acuity were uploaded to the cloud-server. If the images were not of sufficient quality to grade for disease, about 8% of cases, a notation stating this was recorded in the report and the patient was recommended to see an eye care provider within-3 months. Concurrently, an e-mail notification that the report was available was sent to the clinic’s contact person. Once downloaded from the web portal it was added to the electronic medical record (EMR). These results and recommended follow-up duration for an eye doctor visit were shared with the patient via phone within a few days. Patients did not get billed for this program.

### Participants

Participants were recruited in 2017 using convenience sampling from the 2 primary care clinic settings. Eligible participants were identified by clinic staff through a review of EMRs as having diabetes and being someone who would be medically and cognitively fit to interview or participate in a focus group conducted in English. They also either had a dilated eye exam, been assessed via teleophthalmology, or had not seen an eye doctor in at least the last two years. The clinic staff asked eligible participants if they would be interested in the study either in person or by phone. Interested participants were contacted via phone by the study staff to schedule a convenient time for a focus group or an interview, but not both. Eligible participants were at least 18 years old and had diabetes. Individuals were excluded if they did not speak, read and write English, or reported that they were legally blind when asked during a phone screening interview. The focus groups and interviews were conducted in English and participants needed to be able to see how a digital camera could take a picture of the retina as depicted in an on-line video.

Out of the 90 patients identified by the clinic who were reachable and eligible for a prescreening phone interview, 42 agreed to participate, and 23 participated and completed the study. Based on their utilization of eye exam, participants were categorized into the following groups: experience with teleophthalmology (n = 7) or no experience with teleophthalmology (n = 16). A third group (those who had not had a dilated eye exam in the last two years and had not had experience with teleophthalmology) was identified by clinic staff as potential subjects, but none of these patients participated in a focus group or were interviewed when asked. Detailed methods are reported using the COREQ checklist [32].

### Data collection

Semi-structured interviews and 2 focus groups were conducted from April to July 2017 by a facilitator and a research assistant in the two primary care settings, participants’ homes, or another location as preferred by the participant. The focus groups and interviews were conducted by two female master’s degree holding doctoral students in human development who had experience conducting focus groups, interviews, and performing qualitative and qualitative assessments in previous clinically oriented research studies. There was no prior relationship between the focus group or interview facilitators and study participants. At the start of the focus group or interview, the facilitators discussed the study purpose, their credentials, and role. Family members of patients could be present but could not participate in the focus groups or interviews.

Each interview/focus group lasted approximately 45–60 minutes, facilitated by an interview guide (on-line supplementary appendix S1 Text). Participants agreed to be audio-recorded. These were transcribed by a professional transcriptionist. Transcriptions and field notes taken during and after the focus groups and interviews were used in the data analysis. Data collection continued until data saturation was reached.

In both the interviews and focus groups, participants first completed a brief (~10-minutes) self-administered survey [All relevant data underlying this study are within the paper. The full survey data can be found at https://doi.org/10.5281/zenodo.3550069] in English with open and closed ended items that were derived from the behavioral risk factors survey study [33] and previously published literature on the perception and satisfaction of teleophthalmology programs and obtaining dilated eye exams among patients with diabetes [31], but was not pilot tested. The survey including the following sections and is detailed in supplemental material: Demographic information (7-items), health information (6-items), personal views on the importance of eye care, having a dilated eye exam (5-items), and perceived value of teleophthalmology (9 in dilated exam group and 7-items in teleophthalmology group) (S1 Text). Before completing the teleophthalmology focused section, participants were shown a 3-minute video (https://youtu.be/URqAoD3oap4) on teleophthalmology based examination for diabetic retinopathy similar to the program implemented for our population. They were informed that 1) the intervention served as a limited examination to promptly detect eye disease with diabetic patients, and that 2) it did not replace a comprehensive diabetic eye exam that they would receive from an eye doctor but was a recommended alternative if they could not or had not seen an eye doctor for a dilated eye exam in the past year. Participants who experienced teleophthalmology completed two questions specific to their experience.

Participants in the dilated eye exam only group (i.e., no experience with teleophthalmology) were asked about their comfort with using teleophthalmology if it were to be offered by their primary care office and if they would ask their PCP about the teleophthalmology program. Both groups were asked “if you had to pay for the camera-screening out of pocket, how much would you be willing to pay?”

Upon completing the survey, responses to sections on personal views and perceived value of dilated eye exams and teleophthalmology were the basis for discussion among participants where they shared their views with the group. While the survey responses for willingness to pay (WTP) had specific dollar values corresponding to the standard insurance co-pays for the local patient population seen in the clinics, subsequent discussion elicited more detail on what participants were willing to pay.

### Data analysis

Participant demographics were analyzed using means and standard deviation for continuous variables and frequencies and percentages for categorical variables. Group differences were assessed using ANOVA for continuous variables and chi-square for categorical or Fisher’s exact for smaller sample groupings. A p-value of less than 0.05 was considered to be statistically significant. SPSS (version 24) was used for quantitative data analysis.

Open-ended questions and transcribed data from focus groups and interviews were coded using thematic analysis by two of the authors (RSR, SY). This process involved identifying passages linked to the questions asked in sections 3 and 4 of the survey. First, each coder individually evaluated each response on a line-by-line basis circling key phrases that corresponded with patient perspectives pertinent to the discussion. Then the coder looked for how they were grouped by relevant themes. After the individual coding process, the coders met and reviewed each theme for agreements/disagreements. The disagreements were addressed by going back to the data and recoding it as a group.

## Results

### Participant characteristics

Participant characteristics are shown in Table 1. The 23 participants all had physician diagnosed type 2 diabetes. Seven had undergone teleophthalmology to assess for diabetic retinopathy in their primary care provider’s office (teleophthalmology group). The dilated exam only group consisted of 16 participants who only had a dilated eye exam with an eye doctor to check for diabetic retinopathy. The teleophthalmology group was slightly younger (p < .01), more likely to be employed (p < .05), and less likely to have an eye doctor (p<0.02). Half of both groups reported some difficulty with distance vision, even with glasses. The majority in both groups also reported trouble with reading while wearing reading glasses.

**Table 1:** *Participant characteristics (N = 23)*.

Variable Name	23 Total *N* (%)	Teleophthalmology Group– 7 Total *N* (%)	Dilated Eye Exam Only Group– 16 Total *N* (%)
Mean Age (years) (range) (SD)	56.30 (36–69) (8.0)	50.57 (36–55) (6.6)	58.81 (42–69) (7.5)
Gender			
Male	11 (47.8)	3 (43)	8 (50)
Female	12 (52.2)	4 (57)	8 (50)
Race			
African American	13 (56.5)	4 (57.00)	9 (56)
White	8 (34.8)	1 (14.0)	7 (44)
Other	2 (8.6)	2 (29.0)	--
Employment			
Yes	6 (26.1)	4 (57)	2 (12.5)
No	17 (73.9)	3 (43)	14 (87.5)
Health Insurance			
Yes	22 (95.7)	7 (100)	15 (93.8)
No	1 (4.3)	--	1 (6.3)
Primary Health Insurance			
Commercial	5 (22)	3 (43)	2 (13)
Medicaid	8 (34)	2 (29)	6 (37)
Medicare	5 (22)	1 (14)	4 (25)
No Response	5 (22)	1 (14)	4 (25)
Eye Care Coverage			
Yes	19 (82.6)	6 (85.7)	13 (81.3)
No	2 (8.7)	1 (14.3)	1 (6.3)
No response	2 (8.7)	--	2 (12.5)
Eye Doctor			
Yes	15 (65.2)	2 (28.6)	13 (81.3)
No No response	5 (21.7) 3 (13)	2 (28.6) 3 (43)	3 (18.8) --
PCP Visit			
Less than a year	21 (91.3)	6 (85.7)	15 (93.8)
1 to 2 years	1 (4.3)	--	1 (6.3)
No Response	1 (4.3)	1 (14.3)	--
Last Eye Doctor Visit			
Less than a year	12 (52.2)	3 (43)	9 (56.3)
1 to 2 years	10 (43.5)	3 (43)	7 (43.8)
No Response	1 (4.3)	1 (14)	--
Last Dilated Eye Exam			
Less than a year	11 (47.9)	2 (28.4)	9 (56.2)
1 to 2 years	10 (43.5)	3 (43)	7 (43.8)
More than 2 years	1 (4.3)	1 (14.3)	--
No Response	1 (4.3)	1 (14.3)	--

*Note*: SD = Standard Deviation

### Main results

Tables 2 and 3 compare the results of open-ended written responses and the subsequent discussion including barriers to obtaining a dilated eye exam, the benefits of teleophthalmology, potential barriers to receiving teleophthalmology, and each participant’s WTP for the teleophthalmology service. The reported results are aggregated as responses were similar between the two groups.

**Table 2:** Perceived barriers to receiving dilated eye exam and perceptions of teleophthalmology.

**Perceived Barriers to Obtaining a Dilated Eye Exam with an Eye Doctor**	
**Response Category**	**Representative Quotes From Discussion**
Not a Priority/Not Motivated	‘…one more thing I can procrastinate away for awhile.’ (Patient 22) Good to get check ‘to see where you stand now’ ‘but not extremely important’ (Patient 13)
Cost	‘…if you can’t afford to go… Well you can’t get blood out of a turnip. So what I don’t want that headache. You know I don’t want them trying to send me to collections…’ (Patient 8)
Difficulty Scheduling Eye Exam	“[Can call for an appointment, but] when you get it is a whole another story. Like the eye doctor I use is booked three months in advance so I can make the appointment today but I ain’t gonna see him for three months.” (Patient 14)
Asymptomatic	‘..I see pretty good … go to all my appointments except physical therapy and eye exams…’ (Patient 20)
Transportation Challenges	‘…biggest thing with me was the car. How do I get there and how do I get home…’ (Patient 23) ‘ . . .I didn’t feel comfortable driving in that narrow, constricted area like that with eyes that had been dilated.’ (Patient 9)
**Perceived Benefits of Teleophthalmology Exam at a Primary Care Provider Office Visit**	
**Response Category**	**Representative Quotes from Discussion**
Quick/Convenient	‘Convenience. Don’t have to make two separate appointments to go to two separate places.’ (Patient 7)
Educational/Early Detection	‘Very helpful. First to see if I have the problem they was talking about and then if I did to see what they can do to help me.’ (Patient 15) ‘I found it fascinating cause people don’t realize how important that little ball is in their life,especially being diabetic.’ (Patient 6)
Gives Peace of Mind/Good Experience	‘I mean it keeps us free of mind you know like you feel that your eyes are good I mean to avoid you know blindness and stuff so it’s very important.’ (Patient 3)
Done in a Trusted Setting	‘It’s a lot harder going to a stranger and having to explain maybe they don’t understand as much as somebody here [PCP office] would.’ (Patient 5)
**Perceived Barriers to Receiving Teleophthalmology Exam during a Primary Care Provider Office Visit**	
**Response Category**	**Representative Quotes from Discussion**
Cost	‘Cost. The only thing is if it was like hundreds of dollars then I’d say I’ll wait to go see my eye doctor.’ (Patient 21) ‘… it’s the cost. I mean if you don’t know what the cost is, what your insurance covers…if it’s gonna be covered, it’s free, get it…Everybody says I don’t know what my insurance covers . . .’ (Patient 10)
Prefer to See Eye Doctor	‘I would still like to remain at my regular eye doctor.’
Teleophthalmology Not Available	‘Them not having the camera.’ ‘..machine being broke’ (Patients 8 & 9)
Missing an Appointment	‘I miss an appointment.’ ‘Just not showing up.’ (Patients 1 &2)
Poor Customer Service	‘Bad customer service. That’s the only thing probably would stop me.’(Patient 3)

*Note*: n/a: Participants discussed more topics than they listed as their written responses on the survey they filled prior to the facilitated discussion.

**Table 3:** *Willingness to pay for teleophthalmology exam during a primary care provider office visit*.

Amount Selected	Number of Participants	Representative Quotes from Discussion
$0	3	‘Zero. Cause that’s all I got. Zero. I ain’t got no money.’ (Patient 3) ‘I know how important it is…would be willing to pay what I’m used to paying like a copay, 20 dollars.’ (Patient 20) ‘I’m tight on budget…I try to squeeze $30 out…This is very important to me cause I don’t like borrow money and I don’t…’ (Patient 19) ‘40 or maybe more cause with the three minutes that I’ve seen about it, it seems real important to have.’ (Patient 11)
$10	1	
$20	4	
$30	5	
$40	9	

#### Barriers to obtaining dilated eye exams

Using surveys followed by facilitated discussion allowed for richer and more varied responses. During the discussion, almost all participants strongly voiced the lack of insurance coverage for medical care, being on a fixed income, and having a limited budget as barriers to obtaining a dilated eye exam. Cost of care and the cost to access care were main themes in all interviews and focus groups. The discussion also highlighted two additional barriers: transportation challenges and being asymptomatic. Participants commented on the difficulty of convenient parking and safety driving post dilation. Many also spoke about ‘forgetting to make an appointment’ or ‘putting off making an appointment’ especially if they did not have visual or eye symptoms.

#### Value of a teleophthalmology exam

Participants listed convenience (48%) and the ability to detect disease early to give oneself ‘peace of mind’ by knowing and being educated on the status of one’s eye health (35%) as reasons to have a teleophthalmology exam at their primary care visit. The value of teleophthalmology included its quickness and convenience, a ‘one stop shop.’ In addition, participants acknowledged value in not only giving reassurance that there was no vision threatening retinopathy but also in allowing for early detection of disease so that ‘something could be done about it’ to allow for potential treatment to prevent vision loss. Personalized education from having the provider review the findings in the retinal photos to understand the disease better was also of value.

Most respondents reported their WTP as the amount of their usual visit copay for the teleophthalmology exam, but actual costs for the exam were not discussed. More than half indicated that they would be WTP $30 or $40 for the teleophthalmology service on their survey. In this small sample, there was no significant relationship between WTP and type of health care insurance, eye care coverage, or employment status. (Table 3)

While missing primary care appointments or ‘not showing up’ and potential ‘poor customer service’ were noted as potential barriers by a few participants, everyone focused on cost of care as the primary barrier during the discussion. Many emphasized that they would ‘want to know the cost’ of the teleophthalmology examination before deciding to have it done. Participants would be more likely to participate if they knew that their insurance would pay for the service as they were ‘tight on budget’ and living ‘dollar to dollar.’ Despite noting limitations in what they could actually afford, participants expressed value for having eye exams to ensure good vision with a few stating they would ‘pay $100 to $200’ for an exam ‘if [they] could afford it.’

The overall experience of participants who had a teleophthalmology exam was positive. They expressed confidence in primary care staff skills for conducting the examination and labeled it as a ‘helpful service.’ Teleophthalmology fit well in their primary care visit and many stated it was an ‘excellent experience.’ They would recommend teleophthalmology to a friend and would be willing to have such an exam again. In addition, everyone in the dilated exam only group, noted that they would be ‘comfortable’ with having a teleophthalmology based examination at their PCP office.

Although three (13%) said they would prefer an in person dilated eye exam with an eye doctor over a teleophthalmology exam, 20 participants (87%) expressed interest in having a teleophthalmology exam at their PCP office if it was recommended by their PCP.

## Discussion

Using a qualitative approach, we found that a low income, urban, largely African American sample of patients with type 2 diabetes greatly valued having PCP based teleophthalmology, would recommend such a service, and were willing to pay at least the amount of their usual copay. Cost was an important influencer of value. We are the first to report on WTP as an indicator of the perceived value of teleophthalmology to patients. Our study also highlights the importance of having a facilitated discussion to qualitatively assess knowledge, beliefs, and attitudes among US low-income predominantly African American patients with diabetes as such discussion allowed for richer and more varied responses than surveys requiring participants to answer questions on their own.

We not only identified many of the same barriers to obtaining a dilated eye exam as other US based studies [18–20, 31, 32, 34, 35], but also demonstrated the value of a teleophthalmology service using nonmydriatic retinal cameras in PCP practices in overcoming such barriers. The most common stated value was convenience and the ability to overcome transportation and time management issue, as noted in other international and US studies, including a recent study of a white Caucasian rural population in Wisconsin [21, 23, 27, 30, 31, 34, 35]. Other value included ease of use, ability to detect disease before visual symptoms, and the knowledge provided by the photos and technicians about retinopathy and eye disease, which have been only reported thus far in international studies [21–27]. In addition, the use of nonmydriatic cameras without dilating the eye and avoiding temporary vision impairment was seen as a major advantage of teleophthalmology as noted in the recent Wisconsin study [30]. PCP recommendation and stronger PCP-patient relationships were important patient motivators for using teleophthalmology, similar to other studies from Norway [21] and Wisconsin, USA [20, 30].

The cost of care was the major barrier to obtaining dilated eye exams, as seen in other US based studies [16, 18–20, 31, 34–37]. Cost was universally cited as a potential barrier to obtaining a teleophthalmology based nonmydriatic camera exam even if conveniently offered in the primary care office. Educating patients on the potential costs and value of having a teleophthalmology based examination versus going to see an eye doctor for a dilated comprehensive eye exam may be helpful to encourage informed discussions on eye care especially in low income, underserved populations. Combining patient preferences and WTP can provide a more holistic picture of value for a health service such as teleophthalmology by incorporating economic evaluations, such as cost-utility analysis [38]. A recent systematic review of economic studies demonstrated increased cost savings for using teleophthalmology for retinal screening in patients with diabetes versus traditional exams with an eye doctor especially in populations with a higher prevalence of diabetic retinopathy, including minority, low income groups included in our study [39].

While teleophthalmology was well received, there were some who expressed a strong preference to see their eye doctor. These individuals were among the older ones in the group. They expressed valuing their relationship with their eye care provider and questioned the level of expertise and thoroughness of exam afforded by the primary care based teleophthalmology, a finding similar to a recent study of US Veterans [31]. Thus, ensuring that patients, especially older adults, are comfortable with the quality and reliability of teleophthalmology is important. A recent study among American Indians demonstrated that although the digital divide may be greater among low income minority groups, younger American Indian adults were more familiar with digital communication and technology and may be more apt to adopt such methods for accessing health care [40]. The participants in our study who had experienced teleophthalmology were also younger than those who just has a dilated eye exam, which may also have influenced its overwhelming acceptance in our study.

Strengths of our study include having feedback from those who have used teleophthalmology to evaluate their eyes for diabetic retinopathy. It is also the first known to ask a potential customer’s willingness to pay (WTP) for the teleophthalmology service. Consumer WTP has been studied for other telemedicine services, especially teledermatology, whose store and forward model is similar to the present one used for teleophthalmology [41]. Quicker and more convenient access to the expertise of a dermatologist with increased chance of receiving an accurate diagnosis in a timely fashion were related to higher WTP [41, 42]. Furthermore, the use of a pre-discussion questionnaire followed by facilitated dialogue in our study allowed for richer and more varied responses than either option alone. While many studies have looked at attitudes, beliefs and knowledge around eye care and having a dilated eye exam, especially for underserved US populations, our study is among the few to provide insight on the value and perceptions of teleophthalmology in US low income, minority populations.

Limitations of this study include factors pertaining to the composition of our focus groups and interviews and the use of convenience sampling. We also restricted our population to English speakers who were not legally blind. The small number in the teleophthalmology group limited statistical comparisons. Interpreting our participants’ WTP should be done while considering that all participants expressed the importance of an eye exam and had sought eye care within the last two years. We also chose to ask if participants were WTP discrete values from $0 to $40 in our pre-discussion survey, which may have limited our ability to elicit a full range of WTP values. However, encouraging dialogue around their WTP during the discussion found participants’ WTP ranging from $0 to $100–200. Moreover, WTP and what one actually pays may not be the same [43].

Although teleophthalmology was universally seen as valuable by our participants, cost remains a formidable barrier to obtaining such care and to widespread implementation as recently reviewed by Liu at al. [44]. The issue of cost as a barrier to using teleophthalmology for patients and clinics appear to be unique to the US due to its diverse fee for service insurance system, with the exception of the Veterans Affairs Health System. A review of European studies using teleophthalmology to screen for a variety of eye conditions demonstrated substantial cost savings to their national health systems [45]. However, a review of the current state of teleophthalmology in the US by Rathi et al. noted significant gaps in insurance coverage for teleophthalmology among private and government insurers [46]. Further research to test the relation between a population’s price sensitivity and their value for convenience and other benefits provided by teleophthalmology to remotely diagnose eye disease is needed. In addition, the impact of various billing models, including value based and fee for service payments, on the adoption and sustainability of teleophthalmology should be explored. Such research will better elucidate the value of teleophthalmology and help support its use in non-eye care settings for various subsets of potential users.

## Supporting information

S1 Text Outline of pre-focus group questionnaire administered in preparation for focus group discussion. (DOCX)
