# Supplementary material for: Patient perceived value of teleophthalmology in an urban, low income US population with diabetes
Source: PLoS One. 2020 Jan 9;15(1):e0225300. doi: 10.1371/journal.pone.0225300 (PMC6952085; doi:10.1371/journal.pone.0225300)
Supplement: S1 Text — (DOCX) [file pone.0225300.s001.docx]

Outline of Pre-focus Group Questionnaire Administered in Preparation for Focus Group Discussion

DEMOGRAPHIC INFORMATION

Participants were asked about their gender, age, race/ethnicity, employment, health insurance, and if they had any kind of health insurance coverage for eye care. All items except ‘age’ included categorical responses. Participants were asked to write down their age.

HEALTH INFORMATION**:**

Participants were further asked about how they felt their current vision at distance was and near and their utilization of and access to eye care. Participants were asked to report “When was the last time you saw your eye and primary care doctor and had your eyes dilated for an eye exam?” The response options were: Less than a year, 1-2 years, 3-5 years, and 6 years or longer.

PERSONAL VIEWS ON IMPORTANCE OF EYE CARE (5-items)

Participants were asked about their views and concerns regarding their current vision and eye care. In addition, participants were asked to read a scenario about a friend having trouble with their vision and asked respond to questions such as “How important is it for your friend/you to get an annual dilated eye exam?” Response options included, 1 (Not at all) to 5 (Very important). Furthermore, in an open-ended question, participants were asked “What has stopped you from getting a dilated eye exam?”

PERCEIVED VALUE OF TELEOPHTHALMOLOGY BASED EXAMINATION**:** Participants then completed the items about their views about teleophthalmology. Participants who had experienced teleophthalmology completed questions about their experience (e.g., “What was it like to get pictures taken of your eyes at your primary care doctor’s office?” using the following response options: 1 (Poor) to 5 (Outstanding). They were also asked how willing they were to use teleophthalmology again and recommend it to a friend using the following responses: 1 (Not at all) to 5 (Very willing).

Participants in the dilated eye exam only group (who had not undergone a teleophthalmology examination) were asked “If your primary care doctor offered the camera-based screening, how comfortable are you with receiving the camera screening (pictures taken of your eyes) at your primary care doctor’s office?” The response options included: 1 (Not at all comfortable) to 5 (Very comfortable). In addition, the participants in the dilated group were asked “How willing would you be to ask your primary care doctor about the camera eye screening?” and the response options included: 1 (Not at all) to 5 (Very willing). Furthermore, both groups were asked “if you had to pay for the camera-screening out of pocket, how much would you be willing to pay?” and the response options included, $0, $10, $20, $30, and $40.
